# Gene-Based Network Analysis Reveals Prognostic Biomarkers Implicated in Diabetic Tubulointerstitial Injury

**DOI:** 10.1155/2022/2700392

**Published:** 2022-08-31

**Authors:** Sumin Wu, Wei Li, Binhuan Chen, Xuefeng Pei, Yangjian Cao, Yuting Wei, Ye Zhu

**Affiliations:** ^1^Department of Nephrology, Fifth Affiliated Hospital of Sun Yat-sen University, Zhuhai, Guangdong, China; ^2^Department of Pathology, Fifth Affiliated Hospital of Sun Yat-sen University, Zhuhai, Guangdong, China

## Abstract

**Background:**

Diabetic nephropathy (DN), a significant cause of chronic kidney disease (CKD), is a devastating disease worldwide.

**Objective:**

The aim of this study was to reveal crucial genes closely linked to the molecular mechanism of tubulointerstitial injury in DN.

**Methods:**

The Gene Expression Omnibus (GEO) database was used to download the datasets. Based on this, a weighted gene coexpression network analysis (WGCNA) network was constructed to detect DN-related modules and hub genes. Gene Ontology (GO) and Kyoto Encyclopedia of Genes and Genomes (KEGG) enrichments were performed on the selected hub genes and modules. Least absolute shrinkage and selection operator (LASSO) Cox regression analysis was performed on the obtained gene signature.

**Results:**

The WGCNA network was constructed based on 3019 genes, and nine gene coexpression modules were generated. A total of 57 genes, including 34 genes in the magenta module and 23 genes in the purple module, were adapted as hub genes. 61 significantly downregulated and 119 upregulated genes were screened as differentially expressed genes (DEGs). 25 overlapping genes between hub genes chosen from WGCNA and DEG were identified. Through LASSO analysis, a 9-gene signature may be a potential prognostic biomarker for DN. To further explore the potential mechanism of DN, the different immune cell infiltrations between tubulointerstitial samples of DN and healthy samples were estimated.

**Conclusions:**

This bioinformatics study identified CX3CR1, HRG, LTF, TUBA1A, GADD45B, PDK4, CLIC5, NDNF, and SOCS2 as candidate biomarkers for the diagnosis of DN. Moreover, DN tends to own a higher proportion of memory B cell.

## 1. Introduction

Diabetic kidney disease (DKD), also known as diabetic nephropathy (DN) in the past, usually occurs in patients with type 1 and type 2 diabetes mellitus (DM) without adequate long-term glycemic control and is the leading causes of death in patients with diabetes [1]. DN is one of the most important causes of chronic and end-stage renal disease (ESRD) worldwide [2]. In the United States, about 200,000 patients go through ESRD because of DN and 50,000 new patients start dialysis each year [2]. DN patients with ESRD are faced with an approximately 20% annual mortality rate, which is higher than the rate for many solid cancers (including prostate, breast, or even renal cell cancer). The most important of all, the dialytic patients with diabetes have higher mortality than those without diabetes [3]. As a traditional point, glomerulopathy was regarded to play a central role in the progression of DN, while tubulopathy was recently reported as an important diagnosis target in DN [4, 5]. However, the underlying mechanisms of diabetic tubulointerstitial injury in the progression of DN remain poorly understood.

Gene network analysis is a method to find key genes by dividing genes into different modules by the similarity of expression, which helps to systematically understand the gene function at the molecular level [6]. Coexpression networks have been found useful for describing the pairwise relationships among gene transcripts and in identifying the early detection biomarkers and therapeutic targets [7–9]. Until now, various biological processes were elucidated, including cancer [10–12] and noncancerous diseases [6, 13, 14]. Thereby, the weighted gene coexpression network analysis (WGCNA) is a method for the analysis of the gene expression patterns of multiple samples. It can cluster genes and form modules by similar gene expression patterns and analyze the relationship between modules and clinical subtypes [15]. In this study, according to the WGCNA algorithm, the gene expression network should be assumed to follow a scale-free distribution. Within this bioinformatics analysis, gene coexpression networks and a hierarchical clustering tree should be built by estimating the dissimilarity coefficients of different nodes. Moreover, modules should be identified by classifying high similarity genes into the same modules and low similarity genes into different modules, finally identifying the corresponding gene modules for the clinical characteristics.

This research was aimed at revealing DN-related specific hub genes and the respective potentially involved pathways. Based on the Gene Expression Omnibus (GEO) database, gene expression data should be assessed, while subsequently, a coexpression network should be constructed, and important modules with diseases and identification of key genes for further selecting the hub genes were carried out using WGCNA. The results of this study should provide better understanding of the molecular mechanism involved in DN to form a basis for future research to reveal potential diagnostic and/or therapeutic approaches.

## 2. Materials and Methods

### 2.1. Datasets

The microarray dataset GSE104954 including the tubulointerstitial transcriptome from the European Renal cDNA Bank (ERCB) subjects with 105 kidney disease samples and 21 living donor biopsies was downloaded from the GEO database (https://www.ncbi.nlm.nih.gov/geo/query/acc.cgi?acc=GSE104954) [16]. Samples included in this analysis have been previously analyzed using older CDF definitions and are included under previous GEO submissions—GSE47184 (chronic kidney disease samples) and GSE32591 (IgA nephropathy samples). Diabetic nephropathy (DN, *n* = 17) and 21 healthy donors' samples were chosen.

### 2.2. Data Processing

The gene expression information was achieved through microarray information by the expression value of samples from the GEO dataset. Microarray samples were matched with corresponding genes by using annotation information. Samples with more than one gene were eliminated, and the averaged value was calculated for genes corresponding to more than one sample. The level of expression was calculated using variance analysis (12,074 genes), and selected genes with a variance greater than all the quartiles of variance (3019 genes) were selected for further coexpression network construction. Prior to performing the WGCNA calculation, genes and samples with too many missing values were firstly checked by using the goodSamplesGenes function (from WGCNA package) [16]. Then, the samples were clustered to see if there are any obvious outliers, and two clusters without outlier were observed.

### 2.3. Coexpression Network Construction

Firstly, the expression values of 3019 genes in 38 samples were used to construct a scale-free coexpression network using the WGCNA algorithm. The soft-thresholding power was calculated by network topology analysis, and “*sft$powerEstimate*” was chosen as the soft-thresholding power [16]. Subsequently, an adjacency matrix was constructed to describe the correlation strength between the nodes. The correlation between genes was calculated by Pearson correlation matrix and the means of the connecting rod. Then, adjacency to the topological overlap matrix (TOM) was converted, which is a method to quantitatively describe the similarity in nodes by comparing the weighted correlation between two nodes and other nodes. Average linkage hierarchical clustering was performed based on the TOM-based dissimilarity measure to group genes with similar patterns into modules, each containing at least 30 genes (minModuleSize = 30). Finally, the module eigengene (ME) was calculated, which was defined as the first principal component of the expression matrix for a given module, hierarchically clustering and merging similar modules (abline = 0.28).

### 2.4. Identification of Clinically Significant Modules

The module eigengene (ME) was correlated with different disease types to quantify module–trait associations and to discover the most significant associations to determine the interesting modules and clinical traits. Using gene significance (GS, the correlation between the gene and the trait) and module membership (MM, the correlation of the module eigengene and the gene expression profile) helps to quantify associations of genes with DN and to identify genes as candidates for subsequent analysis.

### 2.5. Functional Enrichment Analysis

A Gene Ontology (GO) analysis was performed on the interesting modules, which were relatively associated with clinical features by using the “ClusterProfiler,” an R package for statistical analysis and visualization of functional profiles for genes and gene clusters (https://www.bioconductor.org/packages/release/bioc/html/clusterProfiler.html) [17]. Adjusted *P* < 0.002 was used as the threshold to identify the enriched GO terms and Kyoto Encyclopedia of Genes and Genomes (KEGG) pathways.

### 2.6. Identification of the Hub Genes

The genes with MM > 0.8 and GS > 0.8 in magenta and purple modules, i.e., 34 and 23 genes chosen from magenta and purple modules as hub genes and DEG, respectively, were identified by using the limma package, with log2 − fold change ≥ 1 and *P* < 0.001 as cutoff values (https://bioconductor.org/packages/release/bioc/html/limma.html). The analysis was performed in R language version 3.46.0, and 25 overlapped genes were selected for LASSO regression analysis, which was performed with the glmnet package (https://cran.r-project.org/web/packages/glmnet/index.html) and resulted in 9 genes filtered out [18].

### 2.7. Diagnostic Value and Functional Analysis of Hub Genes

To validate hub genes' diagnostic and prognostic value, ROC analysis was performed by using the pROC [19] package in R and PCA on these genes. At the same time, the PCA plot was visualized by using the ggplot2 package in R [20]. GSVA, a gene set enrichment (GSE) method that estimates variation of pathway activity over a sample population in an unsupervised manner, was utilized on the expression of hub genes to further investigate the potential functions of hub genes [21].

### 2.8. Immune Infiltration Analysis via CIBERSORT

In order to have a deep understanding of the immune microenvironment in DN, the CIBERSORT method was used to analyze the tubulointerstitial transcriptome data. The procession was that the CIBERSORT was applied with a signature matrix file “LM22” at 1000 permutations in the R software and the proportions of 22 immune cells, including naive B cells, CD8+ T cells, naive CD4+ T cells and 19 other kinds of immune cells [22]. Boxplot was used to show the significantly different infiltration level of immune cells between DN and healthy samples by using “ggplot2” and “ggpubr” R packages.

## 3. Results

### 3.1. Construction of Weighted Gene Coexpression Network Identification of Modules Associated with Different Forms of Kidney Diseases

**Figure 1:**
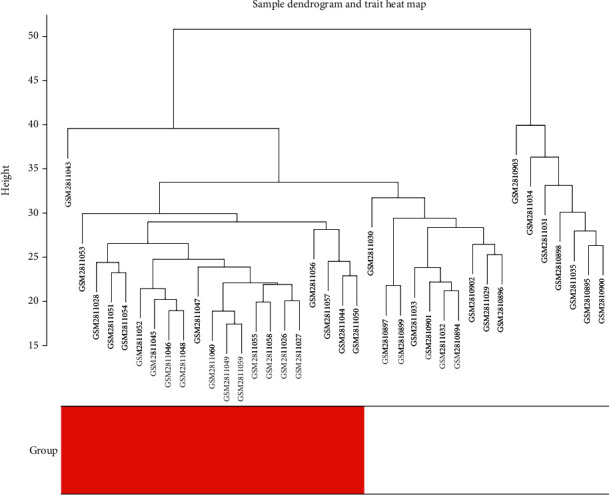
Cluster analysis of samples to detect outliers. All samples are located in the clusters and are divided into two clusters.

**Figure 2:**
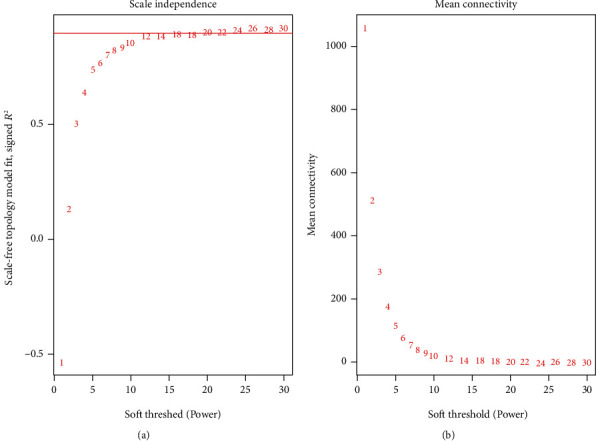
Analysis of network topology for various soft-threshold powers. (a) shows the scale-free fit index (*y*-axis) as a function of the soft-threshold power (*x*-axis). (b) displays the mean connectivity (degree, *y*-axis) as a function of the soft-threshold power (*x*-axis). The red line refers to an *R*^2^ cutoff of 0.9.

**Figure 3:**
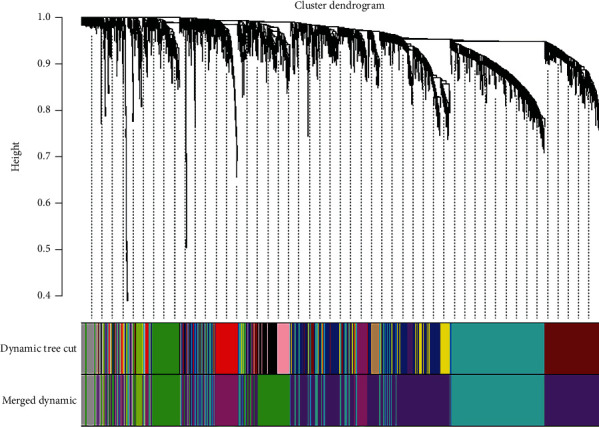
Clustering dendrogram of genes. Clustering based on topological overlap, together with assigned merged module colors and the original module colors. Each leaf, which is a short vertical line, corresponds to a gene. Each color responds to an identified module.

After data processing, genes with variances greater than 75% were chosen for further analysis, achieving a total of 3019 genes. With 3019 genes and 38 samples, samples were clustered to detect outliers by using the hclust function in R and average method (Figure 1), and there were samples that were obviously divided into two clusters. To choose a proper soft-thresholding power in order to meet the criterion of approximate scale-free topology, the function “pickSoftThreshold” was performed, which returns a set of candidate powers. The soft-power threshold *β*5 was determined by the function “sft$powerEstimate” (Figure 2). Using power 5, the adjacencies were calculated, following transformation of the adjacency into a TOM to produce a hierarchical clustering tree (dendrogram) of genes to identify 9 modules. Furthermore, modules whose expression profiles were very similar with choosing a height cut of 0.28 were merged. Finally, 9 modules, ranging from 1279 genes in the purple module to 57 genes in the green-yellow module, were revealed, with an extra module (grey) reserved for unassigned genes, which contained 208 genes (Figure 3).

### 3.2. Correlation between Each Module and Choosing of Interesting Modules

**Figure 4:**
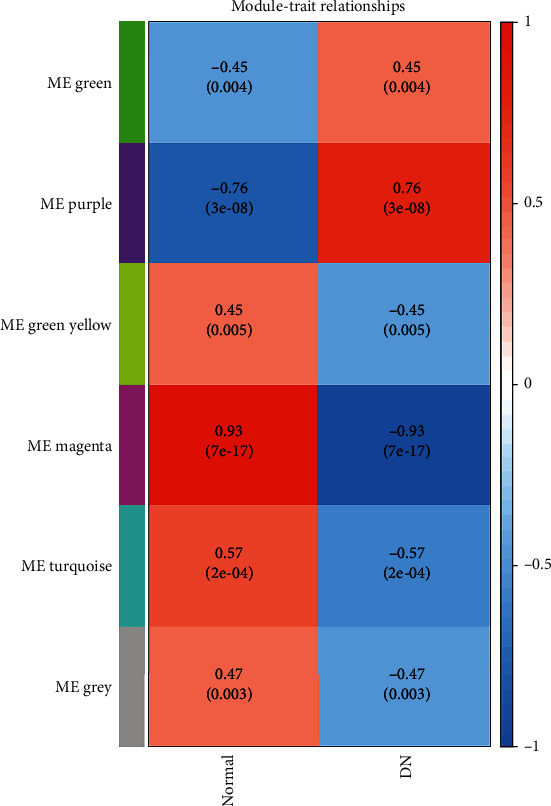
Heat map of relationship between modules and diabetic kidney diseases. Each row corresponds to a module eigengene column to a diseases type. Each cell contains the corresponding correlation and *P* value. The ME values were correlated with binary variables (Spearman's correlation) that represent diabetic nephropathy and healthy cases. Within each table cell, upper values represent correlation coefficients between ME and the variable, while lower values in brackets correspond to *P* value. The table is color-coded by correlation according to the color legend. DN: diabetic nephropathy.

**Figure 5:**
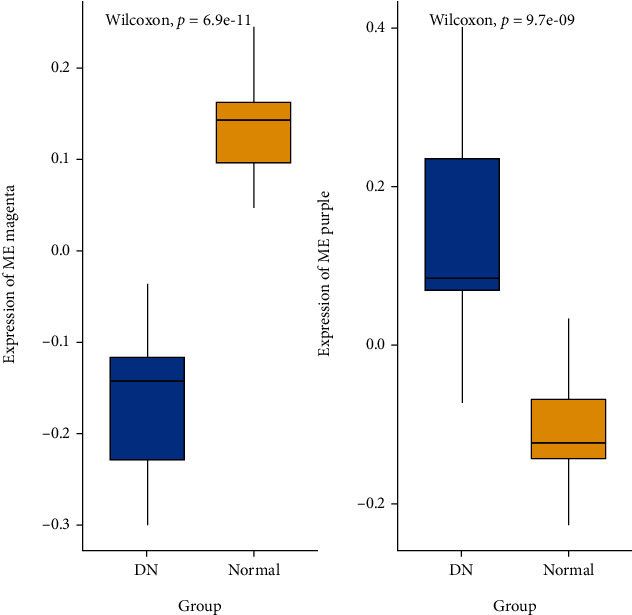
Boxplot containing the distribution of the magenta and purple ME values across the samples. The boxes contain the first and third quartiles; centerline indicates the median and whiskers indicate minimum and maximum values. Kruskal–Wallis test was applied to determine whether ME values were significantly different between the groups.

**Figure 6:**
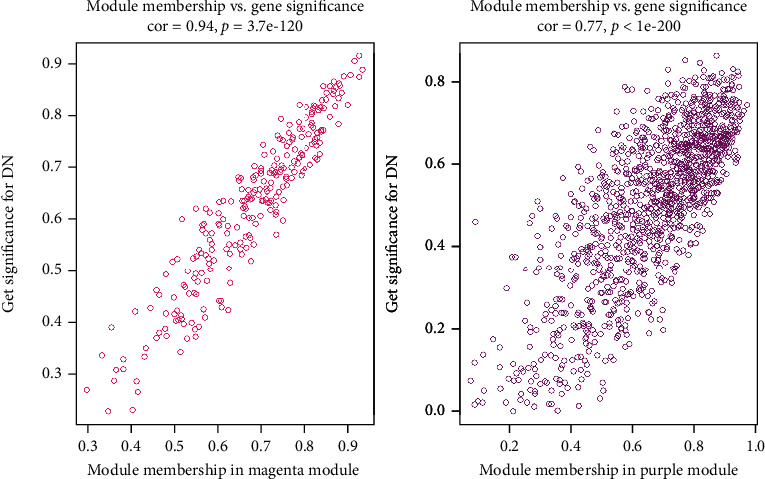
Scatterplot of gene significance (GS) for weight vs. module membership (MM). cor = 0.94, *P* < 0.001 in the magenta module and cor = 0.77, *P* < 0.001 in the purple module.

The associations between modules and DN were quantified by correlating eigengenes in each module with diseases types and visualization of the correlations (Figure 4). Choosing relatively strong associations between clinical traits and modules, box plots were also performed (Figure 5). There were two modules highly related to DN clinical traits, including the magenta (255 genes, *P* < 0.001), which was negatively associated with the disease, and the purple (1279 gens, *P* < 0.001) module, which was positively associated with the disease. In the following analysis, genes that had a high significance for weight as well as high module membership in the two interesting modules were identified by using the GS and MM measures (Figure 6). The two scatterplots, reflecting high correlation between GS and MM, imply that genes significantly associated with a trait were often the most important (central) elements of modules associated with the trait.

### 3.3. Functional Enrichment Analysis

**Figure 7:**
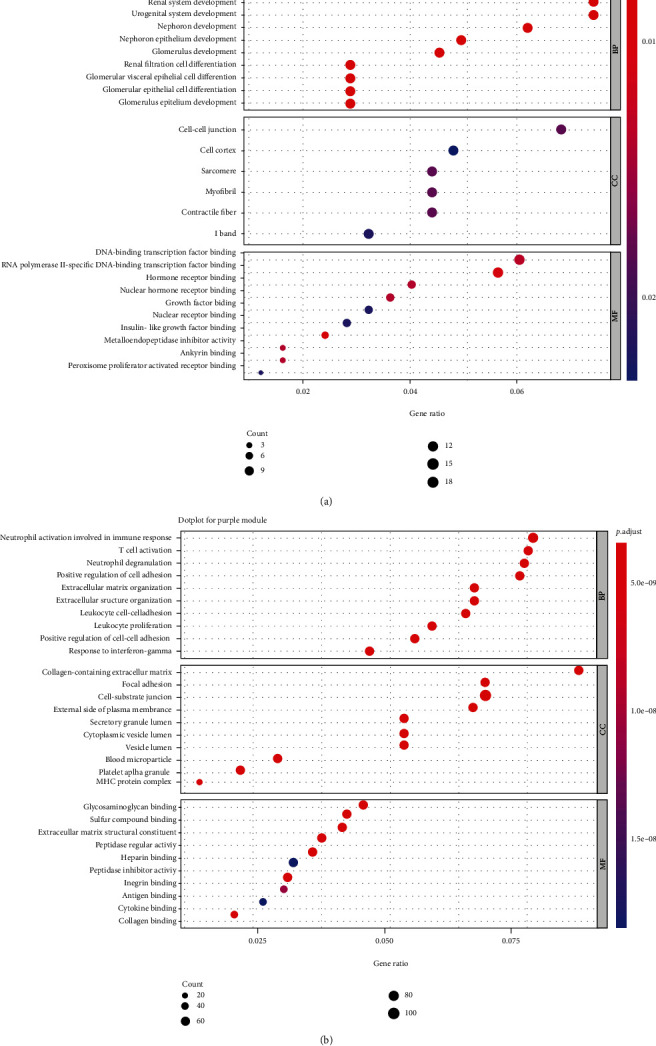
GO analysis on magenta and purple modules. The *x*-axis shows the gene count of each term, and the *y*-axis shows the GO pathway terms. The dot color and size represent *P* value and gene count of each term, respectively. (a) GO enrichment performed on magenta module; (b) GO enrichment performed on purple module.

**Figure 8:**
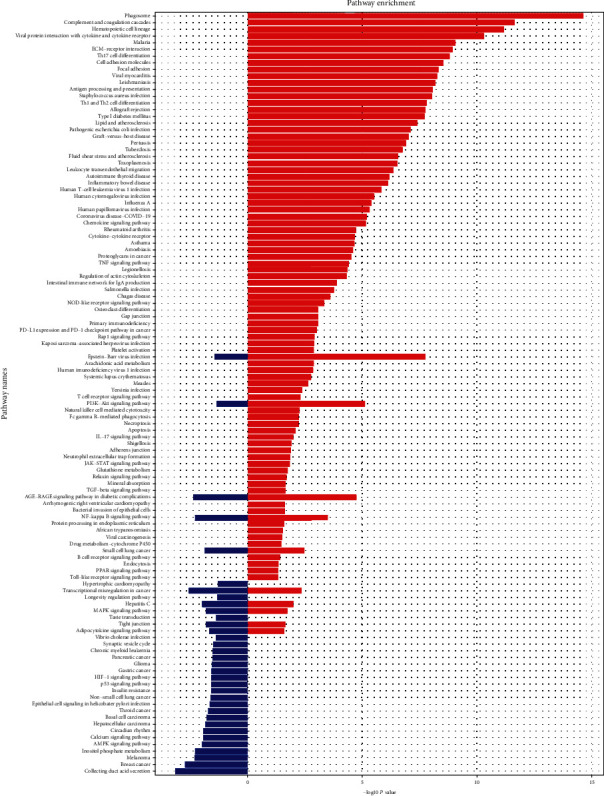
KEGG pathway analysis on magenta and purple modules. The *x*-axis shows the -log10 *P* value of each term, and the *y*-axis shows the KEGG pathway terms. The red and blue colors of the bar represent the purple module and magenta module, respectively.

To explore the potential mechanism of DN and assess prospective functions of the genes within the key module, GO and KEGG pathway analyses were conducted on the magenta and purple modules. Results showed that genes in the magenta module were mainly enriched in kidney development, renal system development, and urogenital system development, whereas genes in the purple module were mainly enriched in “neutrophil activation involved in immune response,” “T cell” activation, and “neutrophil degranulation” (Figure 7). KEGG enrichment analysis showed that genes in the purple module were mainly enriched in the “phagosome,” “complement and coagulation cascades,” and “hematopoietic cell lineage” and genes in the magenta module were mainly enriched in “collecting duct acid secretion” and the cancer-related pathway (Figure 8).

### 3.4. Hub Gene Identification

**Figure 9:**
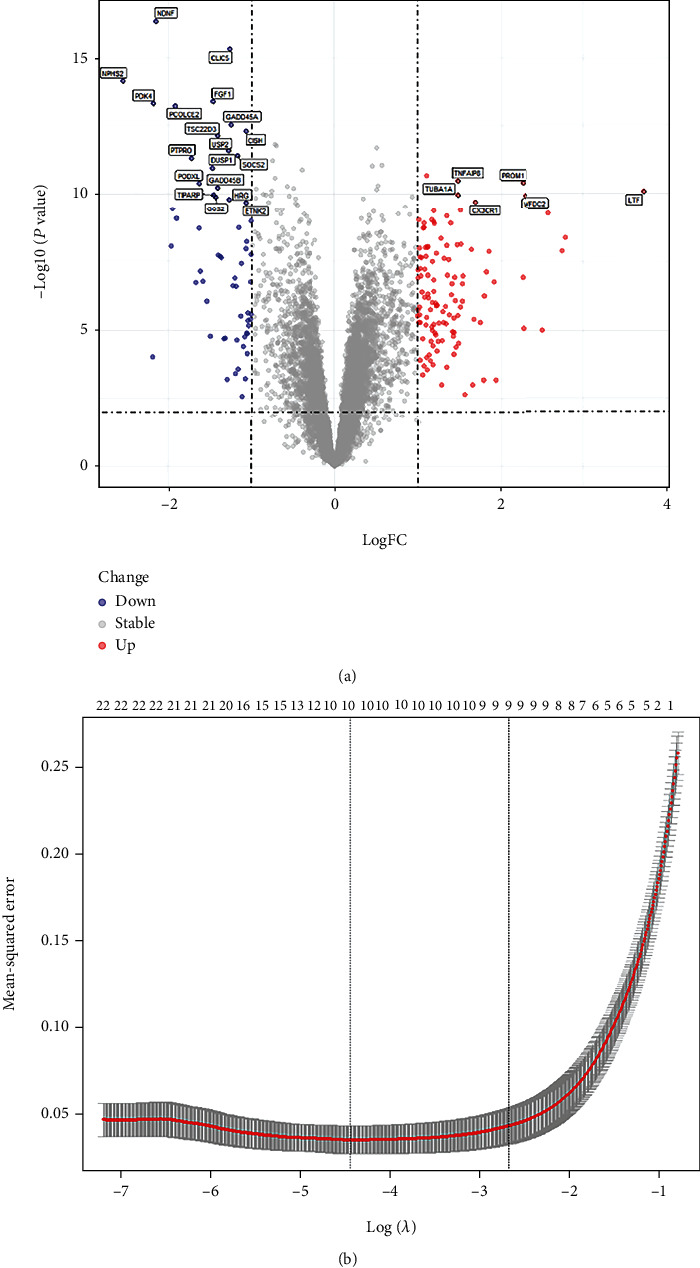
(a) Volcano plot indicating the upregulated and downregulated genes in tubulointerstitial samples from patients with DN. The horizontal axis represents the fold-change between healthy living donors and patients with DN. The vertical axis represents the *P* values of the differences between healthy donors and patients with DN, as determined using Student's *t*-test. The genes most relevant for DN are highlighted in red (2-fold change) or blue (2-fold change). Labels were overlapped genes between DEGs and hub genes choosing from WGCNA. (b) LASSO Cox regression analysis.

Out of the modules, 34 and 23 genes were selected in the magenta and purple modules, which were highly associated with DN and module (GS > 0.8, MM > 0.8). Using a log2 − fold change ≥ 2 and *P* < 0.001 as cutoff values, 180 DEGs were identified in patients with DN, including 61 downregulated genes and 119 upregulated genes. A volcano plot of the log2-fold change vs. the *P* value (-log10 *P* value) for all genes was shown (Figure 9). Finally, 25 overlapping genes were identified. Using 25 overlapping genes associated with DN, the least absolute shrinkage and selection operator (LASSO) Cox regression analysis was utilized, choosing lambda.1se (lambda.1se = 9) as lambda to further choose biomarkers of DN (Figure 9). The results showed that 9 genes were filtered out, including CX3CR1, HRG, LTF, TUBA1A, GADD45B, PDK4, CLIC5, NDNF, and SOCS2.

### 3.5. Diagnostic Value and Functional Analysis of Hub Genes

**Figure 10:**
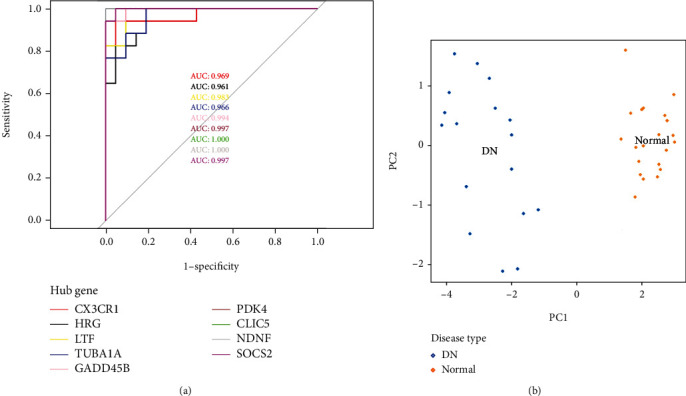
(a) ROC curve of potential biomarkers. Biomarkers include CX3CR1, HRG, LTF, TUBA1A, GADD45B, PDK4, CLIC5, NDNF, and SOCS2. (b) PCA plot of DN and normal samples. The two groups were distinguishably divided into two clusters.

**Figure 11:**
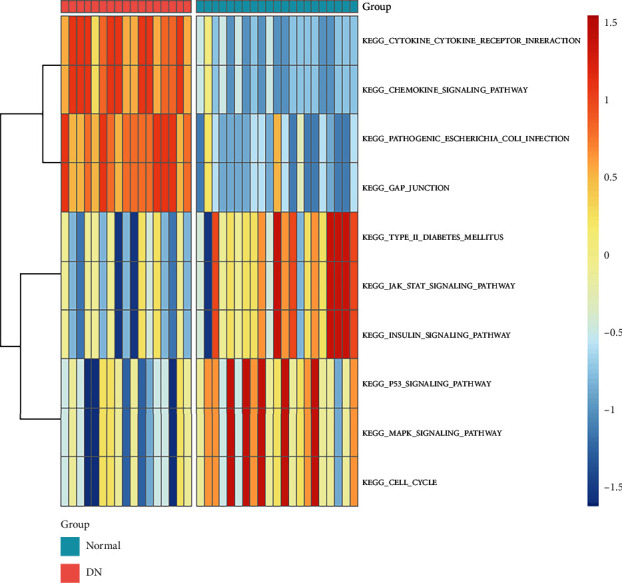
Heat map of the function of 9 hub genes analyzed by GSVA.

ROC curves and area under the ROC curve (AUC) showed their high diagnostic value as biomarkers for DN (Figure 10; CX3CR1 AUC: 0.969, HRG AUC: 0.961, LTF AUC: 0.983, TUBA1A AUC: 0.966, GADD45B AUC: 0.997, PDK4 AUC: 0.997, CLIC5 AUC: 1.0, NDNF AUC: 1.0, and SOCS2 AUC: 0.997). In the PCA plot, DN and normal samples were distinguishably divided into two clusters (Figure 10). These results all showed that these hub genes had good diagnostic values. Moreover, GSVA were performed on these genes to further explore the function of 9 hub genes (Figure 11). Gene sets including CYTOKINE_CYTOKINE_RECEPTOR_INTERACTION, CHEMOKINE_SIGNALING_PATHWAY, GAP_JUNCTION, and PATHOGENIC_ESCHERICHIA_COLI_INFECTION were significantly upregulated in the DN group. However, gene sets including MAPK_SIGNALING_PATHWAY, CELL_CYCLE, P53_SIGNALING_PATHWAY, JAK_STAT_SIGNALING_PATHWAY, INSULIN_SIGNALING_PATHWAY, and TYPE_II_DIABETES_MELLITUS were significantly downregulated in the DN group.

### 3.6. Immune Infiltration Analysis via CIBERSORT

**Figure 12:**
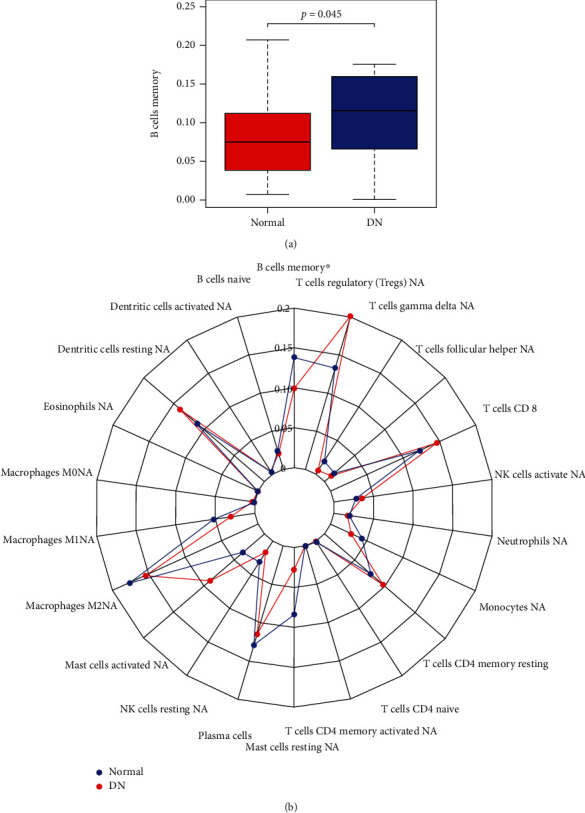
(a) The proportion of memory B cells in normal and DN samples. (b) Radar chart of CIBERSORT.

In order to confirm the role of immune infiltration in the progression of DN, CIBERSORT was performed in the tubulointerstitial transcriptome and immune cells, which were different in DN and healthy samples screened. The results showed that the memory B cell was significantly different between DN and healthy samples. Boxplot and radar chart were plotted to visualize the results (Figure 12).

## 4. Discussion

Diabetic nephropathy (DN) is the leading cause of death in patients with diabetes [1]. Proximal tubulopathy is reported as an important motivator in DN, which is oxygen-deficient because of increased energy demands and reduced perfusion combined with nonhypoxia-related forces resulting in dividing the development of tubular atrophy and interstitial fibrosis [5].

Many factors such as ROS [23], autophagy [24] and inflammation [25] are reported to contribute to diabetic tubulointerstitial injury; however, the mechanisms still remain to be declared. Applying bioinformatics methods including high-throughput microarray help to screen hub genes associated with diabetic tubulointerstitial injury and provide insight into its pathogenesis.

This study constructed a gene coexpression network, which can predict clusters of candidate genes involved in the pathogenesis of DN. It might be hypothesized that tightly coexpressed gene modules, enriched in shared functional annotation, would provide the most fruitful predictions of candidate gene sets that might underlie a given biological process. WGCNA, a package provided in CRAN [26], which provides the WGCNA algorithm to construct the coexpression network and to study the relationship between gene expression and clinical traits, was applied. According to the results, magenta and purple modules were relatively highly associated with DN. GO enrichment showed (1) genes in the purple module, which were highly positively associated with DN, mainly enriched in fibrosis- and inflammation-associated GO terms, such as “T cell activation,” “neutrophil activation involved in immune response,” “collagen-containing,” and “extracellular matrix,” and (2) genes in the magenta module, which were negatively associated with DN, mainly enriched in GO terms involved with the maintenance of normal kidney development and function, such as “kidney development,” “renal system development,” and “urogenital system development.” The results of the KEGG pathway analysis are consistent with the GO enrichment analysis: the purple module is mainly associated with inflammation, such as the “chemokine signaling pathway” and “antigen processing and presentation”; the magenta module is mainly associated with pathways involved in oxidative stress and fibrosis in DN, such as the HIF-1 signaling pathway, AMPK signaling pathway, and p53 signaling pathway [27–29]. According to the results of gene enrichment analysis, the positive module was mainly involved in immune activation and fibrogenesis, whereas the negative module was mainly associated with kidney development and repair. Therefore, it can be hypothesized that genes in the magenta and purple modules may be potential diagnostic biomarkers and therapeutic targets for patients with DN. This appears to be one main implication of the current study.

With threshold values of GS > 0.8 and ME > 0.8, 34 and 23 genes were chosen from the magenta and purple modules, and a total of 180 DEGs were screened using the limma package. For further selection of potential biomarkers of DN, 25 overlapping genes were put into LASSO regression, and finally, 9 genes were determined as candidate biomarkers, including CX3CR1, HRG, LTF, TUBA1A, GADD45B, PDK4, CLIC5, NDNF, and SOCS2. Furthermore, ROC curves and PCA plot showed that all nine genes could serve as biomarkers to distinguish DN from healthy samples sensitively and accurately. Indeed, all these genes appeared as promising candidates as therapeutic targets. C-X3-C motif chemokine receptor 1 (CX3CR1), also known as CCRL1, is identified as a chemokine receptor that selectively targets mouse kidney dendritic cells (DCs), which accumulate in the tubulointerstitium of CKD and produce human transforming growth factor-*β* (TGF-*β*) to drive the development of fibrosis and progression of CKD [29, 30]. Histidine-rich glycoprotein (HRG) is reported to be an activator of ErbB4, which can induce Madin-Darby canine kidney cell tubulogenesis [31]. Roles of lactotransferrin (LTF) are reported by suppressing oxidative stress-induced cell death, protecting against inflammation, augmenting autophagy and antifibrosis in human kidney tubular cells [32]. Tubulin alpha 1a (TUBA1A) can enhance renal tubular cell proliferation and tissue repair but reduces cell death and cell-crystal adhesion to play an important role in kidney stone disease [33]. However, its role in DN remains to be explored. Growth arrest and DNA-damage-inducible 45 beta (GADD45B) participates in mediating cell cycle arrest, DNA damage repair and apoptosis in response to cell injury and has an effect in diabetes-induced renal tubular epithelial-mesenchymal transition (EMT) and apoptosis via the p38 MAPK and JNK pathways, which may be an important mechanism of diabetic kidney injury [34, 35]. The upregulation of pyruvate dehydrogenase kinase 4 (PDK4) drives the mitochondrial dysfunction [36]. More importantly, dysfunctional renal mitochondria serve as pathological mediators of DN, and it is reported that the diabetic milieu and inherited factors that underlie abnormalities in the mitochondrial function synergistically drive the development and progression of DN [37]. Emerging evidence identified chloride intracellular channel 5 (CLIC5) as a new component that is enriched in and necessary for foot process integrity and podocyte function in vivo [38]. Moreover, it has been shown that podocyte injury and loss contribute to the progression of DN [39]. The neuron-derived neurotrophic factor (NDNF) is a glycosylated, disulfide-bonded secretory protein that contains a fibronectin type III domain and suppresses the EMT in RCC cells to inhibit migration and invasion of renal cancer cells [40]. EMT has been reported as a major pathway leading to renal interstitial fibrosis in DN [41, 42]. NDNF may be a protective factor in the generation of diabetic tubulointerstitial fibrosis in DN. Suppressor of cytokine signaling 2 (SOCS2) is a member of the SOCS family, a group of related proteins implicated in the negative regulation of cytokine action through inhibition of the Janus kinase/signal transducers and activators of the transcription (STAT) signal-transduction pathway [43]. Studies show that overexpression of SOCS2 in rat mesangial cells inhibited IGF-1-induced activation of extracellular signal-regulated kinase, which subsequently reduced type IV collagen and DNA synthesis [44].

Moreover, GSVA was applied to the expression of 9 genes to further explore their biological functions. The results of GSVA showed that inflammation-related pathways, such as CYTOKINE_CYTOKINE_RECEPTOR_INTERACTION, CHEMOKINE_SIGNALING_PATHWAY and GAP_JUNCTION were enriched in the DN group of these hub genes, suggesting their contribution to inflammation of tubulointerstitial injury. In order to confirm the role of immune infiltration in the diabetic tubulointerstitial injury, CIBERSORT was conducted in the tubulointerstitial transcriptome, and results showed that memory B cells had a significant difference between DN and healthy tissue. Memory B cells are greatly expanded in kidneys of patients with active lupus, and B cell signaling and activation, lipid/saccharide metabolism and endocytosis pathways were abnormally upregulated in memory B cells [31]. Their possible pathophysiological roles are accelerating apoptosis, poorly costimulating T cells and producing proinflammatory cytokines [45]. In this current study, memory B cells were accumulated in the tubulointerstitial tissue of DN patients. The potential pathophysiological roles of memory B cells in DN remain to be explored.

### 4.1. Strengths and Limitations

This bioinformatics study used a comprehensive analytic protocol to reveal different hub genes, related pathways and immune cells in diabetic tubulointerstitial injury. The findings can provide a good basis for future research in the field. Nevertheless, some limitations must be recognized. The main issue is that the current analysis was restricted to bioinformatics; a validation is still pending and required to reveal the real clinical implications of the findings. In particular, the fact that findings are limited on the transcriptomic level must be taken into account. Additionally, no patient-related information can be considered in such models. Different samples of heterogeneous patients are the basis for the dataset. This limits the ability to draw strong conclusions based on the results. Altogether, the findings are of potential clinical interest; however, further validation of the results is still strictly needed.

## 5. Conclusions

In the current study, 9 genes were screened as candidate diagnostic biomarkers for diabetic tubulointerstitial injury, including CX3CR1, HRG, LTF, TUBA1A, GADD45B, PDK4, CLIC5, NDNF, and SOCS2. Furthermore, DN tends to own a higher proportion of memory B cell. Further research needs to clarify whether the revealed genes lead to new diagnostic and therapeutic targets for patients with DN, for which the current study can form a basis.

## Data Availability

The data used to support the findings of this study are included within the article.
